# Double Disadvantage of Carers with a Disability: A Cross-Sectional Study of Care Duration and Perceived Importance for Service Improvement in Hong Kong, China

**DOI:** 10.3390/ijerph20010020

**Published:** 2022-12-20

**Authors:** Wai Chan, Meiqi Xin, Erin Yiqing Lu, Wai Ming Cheung, Hector Wing Hong Tsang

**Affiliations:** 1 Department of Rehabilitation Sciences, The Hong Kong Polytechnic University, Hung Hom, Hong Kong, China; 2 Mental Health Research Centre, The Hong Kong Polytechnic University, Hung Hom, Hong Kong, China; 3 Faculty of Education, University of Hong Kong, Pokfulam, Hong Kong, China

**Keywords:** carers with a disability, care duration, importance of service improvement, double disadvantage, caregiver assistances

## Abstract

Objectives: this study examined (i) the relationships between the care duration of carers and their perceptions of the importance of service improvement by types of service, and (ii) whether carers had a disability that moderated the impacts of care duration on these perceptions. Design: survey data for cross-sectional analyses. Method: The sample consisted of carers without disability (*n* = 625) and carers with a disability (*n* = 77). Hierarchical multiple regression was applied to examine the unique contribution of care duration. The interaction effects of the disability status of the carer was also estimated. Results: Longer care duration was associated with a greater perception of the importance of service improvement for certain services by carers. The positive relationships between care duration and perception of the importance of caregiver assistances and financial subsidy improvement were stronger for carers with disabilities compared to carers without disability. Conclusions: Long-term carers with disabilities face a potential double disadvantage of service improvement needs with more years of caregiving. Policy makers should consider prioritizing caregiver assistances or financial subsidy service improvements for long-term carers who themselves have a disability.

## 1. Introduction

Caring for a family member with a disability is both physically and mentally demanding, and carers may need to rely on services and community support resources. Recent evidence shows that carers suffer a wide array of health conditions and illnesses, and thus, appropriate services and intervention should be provided to buffer carers from burdens and stresses [1,2]. In fact, it is not uncommon for carers to struggle with access to services due to limited information, long waitlists, poor care coordination, and age eligibility of service users [3,4,5]. Carers often have unmet needs that require service improvement to empower them and their care recipients for better caregiving experiences. 

However, carers’ perceptions of the importance of service improvement have not been fully investigated. It remains unclear whether and how individual differences in carers, particularly their disability status, may account for their perceived importance of service improvement for themselves and their care recipients. To explore service improvement and its associated factors of carers may enhance our understanding of the unmet needs, facilitating the development of intervention programs to address the concerns of stakeholders, and promoting the quality of life of disabled care recipients and their carers [6,7,8]. Addressing service improvement needs should provide us with insights to help allocate resources more efficiently throughout the caregiving process. 

Perceived importance of service improvement may be involved in the subjective assessment of current service utilization and quality [8] and, more importantly, identify unmet needs and ways to improve a given service. However, very few studies have operationalized the importance of service improvement as an outcome of interest. Recent studies on disability service satisfaction only considered some specific types of services, which might miss other vital services needing improvement. For example, Diminic et al. [3] revealed that one-half of carers rated financial assistance, respite care, and emotional support as important services needing improvement. Krevers and Öberg [8] echoed that relief and counseling services were regarded as more important to carers than other services. Dew et al. [9] found that carers reported they had financial concerns during caregiving. These types of service should be further improved and targeted to carers to address their unmet needs. A more comprehensive examination on improvement needs of multiple types of service should be provided. 

### 1.1. Caregiving Load and Service Improvement Needs

Caregiving experiences may hinge on the balance between care demands and support available. Prior studies demonstrated that care duration contributes to caregiving load or burden, but the direction of impacts remained inconsistent. On one hand, carers with fewer years of caregiving responsibility tended to report higher levels of burden and had greater risk of onset of depression [10,11], suggesting that novice carers may experience more psychological distresses than those who have adjusted to care demands. On the other hand, Barnhart et al. [12] revealed that longer care duration was linked with worse general health among caregivers of a family member with mental illness, lending support to the notion of a processes whereby stress accumulates with care duration. Given that studies on care duration in the disability literature are scarce, it would be necessary to further examine its impacts across different subgroups of carers. The potential findings may help to identify the optimal time frame for interventions and service provision to a given group of carers. 

In addition, a growing body of literature suggests that caregiving burden and/or care duration may explain the service improvement needs of carers. Huang et al. [13] found that carers who perceived greater levels of caregiving burden were more likely to use respite care services, suggesting that greater burdens may result in the need for more services. Conversely, more recent carers tended toward institutionalization of their care recipients [14], whereas long-term carers (e.g., more than 10 years of caregiving) reported greater use of respite care [15]. It would be reasonable to assume that care duration might be associated with specific types of service needs. 

### 1.2. Double Disadvantage of Carers with a Disability

It has been well documented that carers have to cope with many challenges on top of their caregiving duties, in which multiple domains of their daily life are often adversely affected, such as health, employment, and social network [2]. Recent studies revealed that past personal experiences or individual differences likely amplify burdens and stresses of carers, which in turn can affect the carers’ well-being and quality of life. For example, Homan and Kong [16] revealed that a mother caring for a child with a disability has a greater risk of poorer health when she was a victim of maltreatment in childhood compared to those who had no history of childhood abuse. Conceivably, when one has to cope with the negative consequences of maltreatment in childhood and the non-normative parenting of a child with a disability, then one’s health and well-being is disproportionately affected or doubly undermined. 

Apart from being a carer, an individual with a disability also has to cope with a great variety of challenges [17]. A few studies reported that people with a disability may face double disadvantages, especially under less favorable or marginalized conditions such as an ethnic minority background or living in a rural area with limited accessibility to services [18,19,20,21,22]. Thus, one would assume a carer with a disability could be experiencing various sources of double disadvantage that would impose multiple challenges on oneself, including the functional limitations of the disability in conjunction with the physical and mental demands of caregiving [17,23,24]. Taken together, non-normative caregiving and the disability status of individuals would likely be subject to double disadvantage among carers of a family member with a disability.

## 2. The Current Study

In Hong Kong, more than 200,000 individuals with disabilities are cared for by carers who assist them in their daily life [25]. This large population of care recipients alludes to the notion that carers do not only look after the care recipients, but they also have to be dependent on themselves. These carers may demonstrate a level of heterogeneity in terms of their individual characteristics, reflected in distinct service improvement needs. The disability status of carers may constitute one of the most important factors in their caregiving experiences. Therefore, the service improvement needs of carers with a disability should be more carefully assessed for more efficient allocation of resources. 

**Figure 1:**
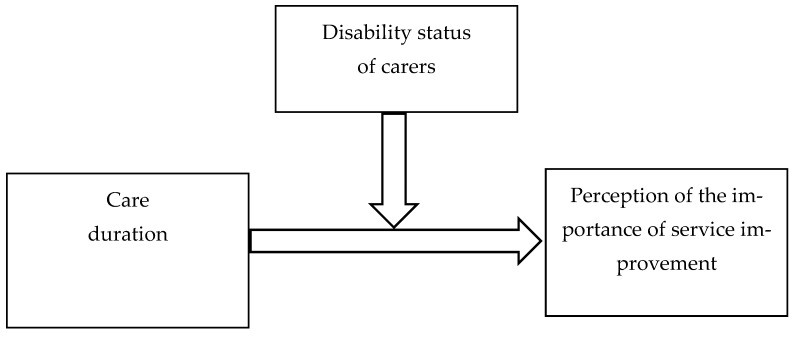
Conceptual model.

Based on the tenets of double disadvantage, the present study aimed to expand the scope of the extant disability research by exploring the relationships between care duration and perceived importance of service improvement. We hypothesized that care duration should be associated with perceived service improvement needs, regardless of the direction of association, due to inconclusive findings from prior studies. Moreover, the disability status of the carer should be associated with higher levels of perceived importance of certain service improvement. To this end, we explored whether the disability status of the carer would moderate the effect of care duration on the perceived importance of service improvement (i.e., double disadvantage effect). It would be reasonable to assume that carers with a disability may exhibit a larger effect of care duration on service improvement needs than carers without disability (see Figure 1).

## 3. Methods

### 3.1. Participants

The current study was based on a subset of quantitative data from a government consultancy project on long-term rehabilitation care in Hong Kong by the Hong Kong Special Administrative Region (HKSAR) led by the corresponding author [26,27]. The project conducted a needs assessment, with the aim to develop recommendations, formulate plans to improve the provision of services, and enhance the quality of life of individuals with disabilities and their caregivers. 

The respondents were recruited through more than 300 nongovernment organizations (NGOs), services units, and self-help associations for people with disabilities across multiple districts in Hong Kong, China (total sample = 1879). Individuals with disabilities living in the community and their caregivers were eligible for the quantitative part of the parent project. The surveys were distributed in paper form between September and October 2019. Responses were collected based on self-report of carers. In the current study, only those caregivers who provided full information were included in the subsequent analyses, giving a final sample size of 702.

### 3.2. Measures

#### 3.2.1. Disability Status of the Carer (1 Item)

The respondent was invited to self-identify whether he or she was an individual with a disability, a carer, or both. Respondents self-reporting as being both a carer and having a disability were coded as 1, whereas those who reported as a carer were coded as 0.

#### 3.2.2. Care Duration (1 Item)

Respondents were asked to indicate the number of years they had cared for an individual with a disability on a 5-point Likert scale (1 = fewer than a year; 2 = 1–5 years; 3 = 5–10 years; 4 = 10–15 years; 5 = 15 years or more). If the respondent provided care to more than one individual with a disability, he/she was asked to report the longest care duration. 

#### 3.2.3. Perceived Importance of Service Improvement (5 Dimensions)

The types of support resources were treated as major outcomes in the current study. The 12 items represent a wide array of services within the local context of Hong Kong (e.g., “to increase the number of District Support Centers for persons with disabilities”, “to increase the quota of Home Care Services”, and “to increase the quota of Day Training Services”) [26,27]. Respondents were asked to rate the importance of each service in terms of the need for improvement on a 5-point Likert scale (1 = not important at all; 5 = very important). A higher value indicates a higher perceived importance in terms of the need to improve that service. 

Based on the service classifications by the HKSAR, the items were categorized into five dimensions: center-based services (5 items; Cronbach alpha = 0.88), home-based services (1 item), respite services (2 items; Cronbach alpha = 0.69), caregiver assistances (2 items; Cronbach alpha = 0.63), and financial subsidy (2 items; Cronbach alpha = 0.78) (see Appendix A). As the internal consistency of these subscales was shown to be acceptable, we calculated the mean score for each dimension. 

#### 3.2.4. Covariates

Four covariates (age, gender, job status, and caregiving support of the respondent) were also included in the analyses. Age was recoded as an interval variable (0 = younger than 10 years to 8 = 80 years or above). Gender (0 = female, 1 = male), job status (0 = currently no job, 1 = having part-time/full-time job), and caregiving support (0 = not having a voluntary caregiver; 1 = having a voluntary caregiver) were entered as dichotomous covariates. 

### 3.3. Statistical Analyses

Several sets of statistical analyses were conducted. First, descriptive statistics were calculated to compare between carers with disabilities and carers without disability. Correlation matrices were constructed to explore the associations between care duration load variables and perceptions of the importance of service improvements at the bivariate level. Second, a series of hierarchical regression models were applied to examine the interaction effects of care duration and carer’s disability. Care duration and other continuous variables (e.g., age) were grand-mean centered to deal with multicollinearity issues. For step 1, main effects of care duration and disability status of the carer were estimated. For step 2, a multiplicative term of care duration and the carer’s disability was entered. If the term was found to be positive and significant, then the disability status may enlarge the association of care duration on the perceived importance of service improvement, yielding evidence to support the double disadvantage hypothesis. Simple slope effects were also examined to explore whether the relationship between care duration and importance of service improvement was significantly different from zero for a given group of carers [28].

## 4. Results

The demographic characteristics of carers with disabilities versus those without disability are summarized in Table 1. About 10% of respondents self-identified as a carer with a disability. Respondents were mostly female, generally aged in their 50s, and typically reported as having no job. Half of the carers with a disability also had a voluntary caregiver, whereas more than half of carers without disability did not have a voluntary caregiver. Both groups of respondents were mostly caregivers for more than 10 years and perceived various types of service as important for improvement.

Correlation matrices are shown in Table 2. Pearson correlation was conducted for major study variables for each group of carers. The positive association between care duration and the perceived importance of certain types of service improvement was dependent on the carer group. Notably, longer care duration was positively associated with greater perceived importance of improvements in center-based services, caregiver assistances, and financial subsidy among carers with a disability at the bivariate level. However, longer care duration was associated with a greater perceived importance of the improvement of respite care among carers without disability. 

**Figure 2:**
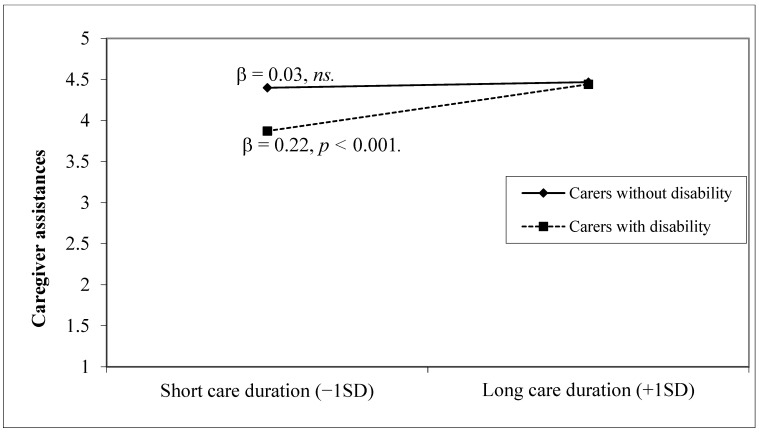
Relationships between care duration and perception of the importance of service improvement among carers on caregiver assistances.

**Figure 3:**
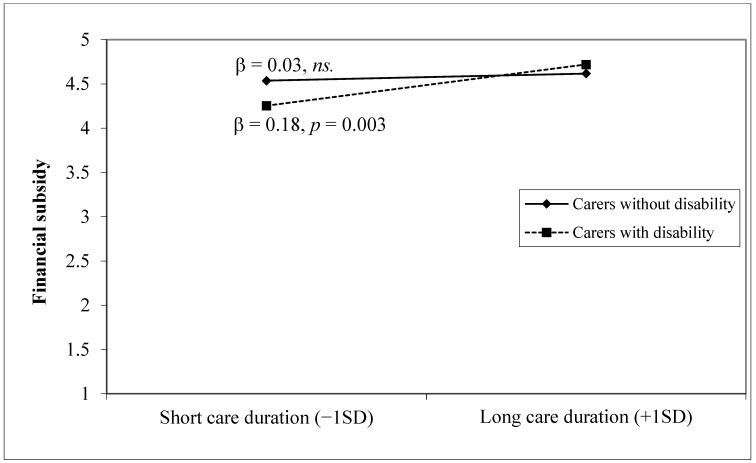
Relationship between care duration and perception of the importance of service improvement among carers on financial subsidy.

To further examine the effects of the disability status of carers on perceived service improvement, we conducted hierarchical multiple regression models (Table 3). For most types of services, longer care duration predicted greater importance of service improvement at step 1, whereas caregivers with a disability perceived less importance in improvement of center-based services, respite services, and caregiver assistances, after adjusting for sociodemographic characteristics. However, at step 2, the moderating effect of a carer’s disability status was detected when perceived importance of improvements in caregiver assistances (Figure 2) or financial subsidy (Figure 3) were entered as outcomes. The simple slope analyses and plots demonstrated there were significant and positive associations between care duration and perceived importance of service improvement only among carers with a disability (βs ≥ 0.18, *p*s < 0.01). In other words, the disability of the carer amplified the positive association between care duration and service improvement needs. These findings suggest that the double disadvantage effects may be conditional on the disability status of carers as a potential risk factor and the importance of certain types of service improvement, providing partial support for the research hypotheses. 

## 5. Discussion

The current study examined the hypothesis that double disadvantage in carers of individuals with a disability in Hong Kong may account for the perceived importance of service improvement, in which the disability status of carers themselves may alter the impacts of care duration on the types of service improvement needs. We found that longer care duration predicted higher levels of perceived importance of service improvements in carers. The positive association between care duration and service improvements in caregiver assistances or financial subsidy was stronger among carers with a disability than those without disability, but such effects were reversed for carers with a short care duration, suggesting that the burden of long-term caregiving and disability status of a carer may act as synergistic effects on service improvement needs. Service improvement needs were not fully exposed among carers with a disability until their caregiving role was assumed for a long period of time. These findings provide additional support for the double disadvantage hypothesis and more information on characteristics of carers at higher risk of service inaccessibility.

A novelty of this study is the expansion of applicability of the double disadvantage hypothesis to the outcomes of service evaluation in the carer population. The double disadvantage hypothesis was developed in the disability literature more than two decades ago, which is often referred to health- or well-being-related outcomes of individuals with a disability [19,20,21,22]. However, service-related outcomes have not been fully examined yet, which plays an important role for enhancing quality of life for both parties of carers and care recipients. Extending previous studies on family context [16], this study sought to add empirical evidence that the effects of double disadvantage may not only apply to health domains of an individual, but it may also account for the perceived importance of different types of service needs. 

The impact of care duration on the perceived importance of service improvements was evident in a few types of service. The results showed the longer the care duration, the greater the perceived importance of service improvement needs. This main effect remained robust for center-based, home-based, and respite services, suggesting long-term carers tended to require greater improvement in multiple types of services. This may be because long-term carers may have already exhausted most services, which may have diminishing utility with increasing care demands and care duration. Some studies also suggested that with longer care duration, carers might be more exposed to the suffering of their care recipients [2]. They may hope for more or better services to cope with different challenges of caregiving as time goes by. Moreover, the majority of carers are older adults with declining physical health or existing health concerns, so these long-term carers may hence try to optimize their caregiving responsibilities by seeking additional or new services to fulfill their caregiving role or to enhance their own well-being. To a certain extent, care duration may emerge as a marker highlighting the need for extra community support for carers. 

Contrary to our expectations, carers with a disability tended to perceive a lower importance of some services, such as center-based services, caregiver assistances, and respite services. Such main effects were robust even after adjusting for other factors. One possibility is that their disability status may exploit their own resources and encourage them from being receptive to most existing services, so they did not desire service improvement as strongly as their counterparts without disability. Another possibility is that they might internalize their caregiving endeavors together with their own disability as a personal misfortune and self-blame [29]. As a consequence, carers with disabilities might not turn to services or community support resources compared to their counterparts without disability. It is not fully clear what their service needs are, or whether and to what extent their service needs have been addressed. It is important to understand whether they are coping with their caregiving responsibilities, and whether their own disability may or may not limit them from seeking additional services. 

Double disadvantage effects were found among carers with disabilities, with synergistic effects between disability status and long care duration that correlated with specific types of service improvement needs. The service improvement needs were found to be more severely affected by disability when the care duration was substantially longer. In other words, these effects appeared to be time-dependent. This may suggest that carers with a disability who have been in a caregiving role for several years are more vulnerable, and they would perceive a greater need for service improvement. This group of carers may have a higher risk of poor physical and mental health on top of the burdens and stresses of their ongoing caregiving responsibilities [30]. Before these additional needs arise, appropriate services should be provided to prevent carers with a disability from health decline and to maintain the quality of life of both carers and care recipients. 

In the current study, double disadvantage effects were identified only on caregiver assistances and financial subsidy outcomes. These effects might be specific to the carer’s needs rather than those of the care recipient. Indeed, prior studies indicated that the needs of carers might contribute more to their service utility than the characteristics of the care recipients [31,32]. On one hand, the caregiving capacity of carers with disabilities might be eroded at a disproportionate rate with care duration and would therefore elicit service improvement needs. On the other hand, financial assistance is often reported as an unmet need and a major source of stress for both carers and care recipients [9,33,34]. This unmet need would likely turn into an appeal for financial subsidy as a service improvement. Such an appeal would more likely come from carers with disabilities than carers without disabilities, based on the current findings. For example, some studies suggested that in mainland China, parents of children with disabilities may develop financial concerns over time as most rehabilitation assistance welfare covers children up to the age of six [35,36]. These parents may have to rely on their own financial resources in the long run, especially when their children would no longer be age eligible for social assistances. Future intervention programs should prioritize service improvement related to caregiver assistances or financial empowerment for longer-term carers with disabilities, considering the relevance of double disadvantage to carers with disabilities [37].

## 6. Limitations and Future Directions

The present study had several limitations that should be considered. First, the sample size between carer groups was not equal, and the group of carers with a disability was not representative of the corresponding subgroup of the carer population in Hong Kong. There were relatively fewer carers with disabilities than carers without disability, which meant the types of disabilities were collapsed to form only a binary grouping factor. This issue is not uncommon in some studies on the effects of double disadvantage [16,21], probably because individuals with multiple challenges are relatively hidden and marginalized in society [18]. Besides caregiving responsibilities, these individuals may be struggling with family obligations and their own physical disabilities, which may restrain them from joining in the study. Hence, the generalizability of the present findings to carers with other conditions may be limited. A voluntary registry system could be feasible to follow-up on a given type of disability and the service needs of carers to ensure appropriate support can be provided. 

Second, compared to well-validated and standardized instruments, the measures in the current study were relatively crude. For example, the disability of respondents documented in the current study was based on self-report of carers, but the diagnosis status and levels of severity were not specified. Clinical assessment and more background information of carers could be incorporated into future studies. Care duration was operationalized as a given domain of caregiving load [15], but a single item may not be able to fully assess caregiving burden. In a similar vein, the instrument used in the current study to explore perceptions of the importance of service improvement did not fully capture the construct of service use or unmet needs. Future studies should consider applying a more comprehensive instrument for the service evaluation outcomes. 

Third, the current study demonstrated a correlation between care duration and perception of the importance of service improvement using only cross-sectional data. It is not entirely clear how caregiving load varies with service utility over time. Service use at multiple time points should be assessed to explore the linearity of the relationship between care duration and service improvement needs. For example, future studies should adopt a longitudinal study design to disentangle how the trajectory of care duration every additional year might change with the service needs and the types of service utilization.

## 7. Conclusions

The current study extended the extant literature on double disadvantage perspective by examining the relationships between care duration and service improvement needs, moderated by the disability status, among carers of a family member with a disability in Hong Kong. The findings showed that longer care duration was associated with greater perceived importance of caregiver assistances and financial subsidy improvement, and such association was amplified among carers with disabilities. The findings implied that carers with a disability were at a clear disadvantage by being more vulnerable to specific service needs than their counterparts without disability when their caregiving duration was substantially long. These carers may be at higher risk for limited accessibility of caregiver assistances and financial subsidy. It is important for service providers and policy makers to consider providing timely intervention to carers at greater risk of unmet needs, which could prevent further impacts of double disadvantage and hence maintain the quality of life of both carers and care recipients. Further studies should also explore how carers with a disability utilize support services and hence should identify their additional service needs, especially for those who have been vulnerable to potential erosion of their caregiving capacity due to the accumulative caregiving duties and their own disability status at the same time. Finally, the present investigation builds on prior studies and highlights the applicability of the double disadvantage perspective to understand the impacts of disability of carers on their caregiving endeavors. To further explore the service needs of carers with constraints other than their disability, this perspective may provide future studies with a useful framework. 

## Figures and Tables

**Table 1:** Sample characteristics (*n* = 702).

Variables	Caregivers without Disability (*n* = 625)				Caregivers with a Disability (*n* = 77)			
	*n*	%	*M*	*SD*	*n*	%	*M*	*SD*
Demographic characteristics								
Age ^a^			4.78	1.25			4.58	1.51
Sex								
Female	492	78.7			56	72.7		
Male	133	21.3			21	27.3		
Having a full/part-time job								
No	432	69.1			54	70.1		
Yes	193	30.9			23	29.9		
Having a voluntary caregiver								
No	384	61.4			35	45.5		
Yes	241	38.6			42	54.5		
Care duration ^b^			3.88	1.34			3.92	1.23
Perceived importance for improvement ^c^								
Center-based services			4.39	0.62			4.11	.81
Home-based services			4.32	0.78			4.21	1.00
Respite services			4.43	0.67			4.07	0.87
Caregiver assistances			4.40	0.64			4.15	0.82
Financial subsidy			4.55	0.63			4.48	0.75
Types of disability			--	--				
ADHD					4	5.2		
ASD					12	15.6		
Hearing impairment					8	10.4		
Intellectual disability					10	13.0		
Mental illness					26	33.8		
Physical disability					21	27.3		
Special learning difficulties					5	6.5		
Speech impairment					6	7.8		
Vision impairment					8	10.4		
Visceral disability					7	9.1		
Others					9	11.7		

Notes: ^a^ 0 = younger than 10 years; 1 = 10–19 years; 2 = 20–29 years; 3 = 30–39 years; 4 = 40–49 years; 5 = 50–59 years; 6 = 60–69 years; 7 = 70–79 years; 8 = 80 years or above. ^b^ 1 = less than a year; 2 = 1–5 years; 3 = 5–10 years; 4 = 10–15 years; 5 = 15 years or more. ^c^ 1 = not important at all; 2 = slightly important; 3 = generally important; 4 = important; 5 = very important.

**Table 2:** Correlation matrices (*n* = 702).

Variables	1	2	3	4	5	6
1. Care duration (centered)	-	0.23 *	0.06	−0.03	0.32 **	0.27 *
2. Center-based services	0.03	-	0.67 ***	0.71 ***	0.76 ***	0.64 ***
3. Home-based services	0.04	0.57 ***	-	0.68 ***	0.53 ***	0.49 ***
4. Respite services	0.16 ***	0.66 ***	0.65 **	-	0.50 ***	0.48 ***
5. Caregiver assistances	−0.04	0.73 ***	0.43 ***	0.49 ***	-	0.74 ***
6. Financial subsidy	−0.01	0.46 ***	0.48 ***	0.51 ***	0.51 ***	-

Notes: Values above the diagonal represented statistics of carers with a disability. Values below the diagonal represented statistics of carers without disability. * *p* < 0.05, ** *p* < 0.01, *** *p* < 0.001.

**Table 3:** Multiple regression models (*n* = 702).

	Center-Based			Home-Based			Respite Services			Caregiver Assistances			Financial Subsidy		
Variables	B	SE	β	B	SE	β	B	SE	β	B	SE	β	B	SE	β
Step 1															
Sociodemographic															
Age (centered)	−0.11	0.02	−0.22 ***	−0.08	0.03	−0.13 **	−0.06	0.02	−0.10 *	−0.13	0.02	−0.25 ***	−0.10	0.02	−0.19 ***
Male	−0.09	0.06	−0.06	0.00	0.08	0.00	0.01	0.07	0.01	−0.11	0.06	−0.07 †	0.01	0.06	0.00
Having a full/part-time job	−0.10	0.05	−0.07 †	0.03	0.07	0.02	−0.09	0.06	−0.06	−0.03	0.06	−0.02	−0.07	0.06	−0.05
Having a voluntary caregiver	−0.04	0.05	−0.03	−0.04	0.06	−0.03	−0.07	0.05	−0.05	−0.00	0.05	−0.00	−0.02	0.05	−0.02
Caregivers with a disability	−0.30	0.08	−0.14 ***	−0.13	0.10	−0.05	−0.37	0.08	−0.16 ***	−0.27	0.08	−0.13 **	−0.09	0.08	−0.04
Care duration (centered)	0.06	0.02	0.13 **	.05	0.03	0.09 *	0.09	0.02	0.17 ***	0.05	0.02	0.09 *	0.05	0.02	0.09 *
*R* ^2^	-	-	0.07	-	-	0.02	-	-	0.06	-	-	0.07	-	-	0.03
Adjusted *R*^2^	-	-	0.06	-	-	0.01	-	-	0.05	-	-	0.07	-	-	0.02
Step 2															
Sociodemographic															
Age (centered)	−0.11	0.02	−0.22	−0.08	0.03	−0.13 **	−0.06	0.02	−0.11 *	−0.12	0.02	−0.24 ***	−0.09	0.02	−0.19 ***
Male	−0.09	0.06	−0.06	0.00	0.08	0.00	0.01	0.07	0.01	−0.10	0.06	−0.06 †	0.01	0.06	0.01
Having a full/part-time job	−0.10	0.05	−0.07 †	0.03	0.07	0.02	−0.09	0.06	−0.06	−0.03	0.06	−0.02	−0.07	0.06	−0.05
Having a voluntary caregiver	−0.04	0.05	−0.03	−0.04	0.06	−0.03	−0.07	0.05	−0.05	−0.01	0.05	−0.01	−0.03	0.05	−0.02
Caregivers with a disability	−0.30	0.08	−0.14 ***	−0.13	0.10	−0.05	−0.36	0.08	−0.16 ***	−0.27	0.08	−0.13 ***	−0.09	0.08	−0.04
Care duration (centered)	0.05	0.02	0.10 *	0.05	0.03	0.09 *	0.10	0.02	0.19 ***	0.03	0.02	0.05	0.03	0.02	0.06
Caregivers with a disability × care duration	0.11	0.06	0.07 †	0.00	0.08	0.00	−0.10	0.07	−0.06	0.19	0.06	0.12 **	0.15	0.06	0.09 *
*R* ^2^	-	-	0.08	-	-	0.02	-	-	0.06	-	-	0.09	-	-	0.04
Adjusted *R*^2^	-	-	0.07	-	-	0.01	-	-	0.05	-	-	0.08	-	-	0.03
Δ*R*^2^	-	-	0.00 †	-	-	0.00	-	-	0.00	-	-	0.01 **	-	-	0.01 *

† *p* < 0.10, * *p* < 0.05, ** *p* < 0.01. *** *p* < 0.001.

## Data Availability

Restrictions apply to the availability of these data. Data were obtained from the LWB, the Government of the Hong Kong, HKSAR, PRC, and data are available from the authors with the permission of LWB.

## Appendix A

	**Center-Based**	**Home-Based**	**Respite Services**	**Caregiver Assistances**	**Financial Subsidy**
1. To increase the number of district support centers (DSCs) for persons with disabilities	☑				
2. To increase the number of parents/relatives resource centers (PRCs)				☑	
3. To increase the number of community rehabilitation day centers (CRDCs)	☑				
4. To increase the number of social and recreational center for the disabled (S&RCs)	☑				
5. To increase the number of support centers for persons with autism (SPA)	☑				
6. To increase the quota of home care services		☑			
7. To increase the quota of day training services	☑				
8. To increase the quota of day respite services			☑		
9. To increase the quota of residential respite services			☑		
10. To provide individuals with disabilities/caregivers with cash subsidies					☑
11. To sponsor individuals with disabilities and families for purchase of assistive technology devices					☑
12. To provide caregivers with emotional support services and disability care skill training				☑	
